# Long-Term Prognosis in Patients with ST-Elevation Myocardial Infarction Complicated by Heart Failure with Preserved Left Ventricular Ejection Fraction

**DOI:** 10.3390/jcdd12070272

**Published:** 2025-07-16

**Authors:** Lidija Savic, Damjan Simic, Ratko Lasica, Gordana Krljanac, Dragan Matic, Milika Asanin, Sanja Stankovic, Nebojsa Antonijevic, Igor Mrdovic

**Affiliations:** 1 Faculty of Medicine, University of Belgrade, 11000 Belgrade, Serbia; drlasica@gmail.com (R.L.); gkrljanac@gmail.com (G.K.); masanin2013@gmail.com (M.A.); drantoni@gmail.com (N.A.); igormrd@gmail.com (I.M.); 2 Cardiology Intensive Care Unit & Cardiology Clinic, Emergency Hospital, University Clinical Center of Serbia, 11000 Belgrade, Serbia; 3 Center for Medical Biochemistry, Emergency Hospital, University Clinical Center of Serbia, 11000 Belgrade, Serbia; sstankovic2013@gmail.com

**Keywords:** ST-segment elevation myocardial infarction, heart failure, ejection fraction

## Abstract

Background/aim: We aimed to analyze eight-year mortality in patients with ST-elevation myocardial infarction (STEMI) complicated by the development of in-hospital heart failure with preserved ejection fraction (HFpEF). Method: We analyzed 3260 STEMI patients treated with primary PCI (pPCI). Reduced EF was defined as value <50% and preserved EF as value ≥50%. Patients were divided in three groups: without HF, with HFpEF, and with HF with reduced EF (HFrEF). Patients with cardiogenic shock at admission were excluded. Results: In-hospital HF was registered in 759 (23.2%) patients. Among the patients with in-hospital HF, 80 (10.5%) patients had HFpEF. Patients with HFpEF had significantly higher 8-year mortality compared with patients without HF (11.2% vs. 3.5%, respectively, *p* < 0.001), but significantly lower mortality compared with patients with HFrEF: 11.2% vs. 25.1%, respectively, *p* < 0.001. In the Cox regression model, HFpEF and HFrEF were independent predictors for 8-year mortality-HFpEF: HR1.85 (95%CI 1.26–4.25); HFrEF: 4.89 (95%CI 3.19–6.42). Conclusion: Development of in-hospital HFpEF in STEMI patients was an independent predictor for long-term mortality. The negative prognostic impact of HFpEF was weaker when compared to the impact of in-hospital HFrEF.

## 1. Introduction

Timely and successful reperfusion with primary percutaneous coronary intervention (pPCI) and subsequent optimal medical therapy are associated with a decreased infarct size, preservation of left ventricular systolic function, and reduced mortality in patients with ST-elevation myocardial infarction (STEMI) [1,2,3,4,5]. It is well-known that left ventricular systolic function is a particularly important predictor of mortality and the occurrence of other adverse events after STEMI [6,7], and that left ventricular ejection fraction (EF) is currently the most commonly used parameter for evaluating systolic function [8,9]. Patients with preserved EF after STEMI are considered to have a low risk of mortality and the occurrence of other adverse cardiac events [2,9,10,11,12,13,14].

Heart failure (HF) complicating STEMI is another strong predictor of mortality and the occurrence of other adverse events. Patients who develop HF are considered high-risk [5,7,10,11,15,16]. HF complicating STEMI is predominantly caused by left ventricular systolic dysfunction (i.e., reduced EF) [10,17,18,19,20,21]. On the other hand, diastolic dysfunction in STEMI patients with preserved EF may also cause HF [9,22,23]. The incidence of HF complicating STEMI is on the decline in the pPCI era, but it has not been completely eliminated, and, according to some authors, it can be as high as 30% [1,3,4,9,10,11,15,17,18,19]. This decline in incidence refers to STEMI patients with heart failure with reduced ejection fraction (HFrEF), while the incidence of heart failure with preserved ejection fraction (HFpEF) as a complication of STEMI has remained stable [18]. Although patients with heart failure with preserved ejection fraction (HFpEF) are generally considered to have better survival than those with HFrEF, most observational studies indicate that this difference is negligible [24]. Results from the large MAGGIC meta-analysis also showed that the adjusted median 2.5-year mortality rate was lower in patients with HFpEF compared to those with heart failure with reduced ejection fraction (HFrEF) [24,25].

Numerous studies have examined the incidence and prognosis of heart failure (HF) complicating myocardial infarction (MI) [4,5,9,10,18]. However, some of these studies included mixed populations of STEMI and non-STEMI patients [9], while others involved STEMI patients treated with various reperfusion strategies, such as primary percutaneous coronary intervention (pPCI), fibrinolytic therapy, or post-thrombolysis PCI [10]. Some registries have specifically evaluated post-AMI heart failure with preserved ejection fraction; however, some of these studies used varying cutoff values to define preserved EF, which differ from current guidelines [26]. There are fewer studies analyzing the prevalence of HFpEF complicating STEMI in the era of primary PCI, and data on the long-term prognosis of these patients are almost non-existent.

This study aims to analyze the long-term mortality in patients with STEMI complicated by the development of HFpEF.

## 2. Materials and Methods

### 2.1. Study Population, Inclusion and Exclusion Criteria, Data Collection, and Definitions

Our study involved 3260 STEMI patients hospitalized between 1 December 2006 and 31 March 2017, who were included in the University Clinical Center of Serbia STEMI Register. The purpose of the prospective University Clinical Center of Serbia STEMI Register has already been published elsewhere [27]. The objective of the Register is to gather data on the management and short- and long-term outcomes of patients with STEMI treated with pPCI. STEMI patients, aged 18 years or older, and without cardiogenic shock at admission were included in this Register. Also, for the purpose of this study, patients with previous myocardial infarction or a previous known structural heart disease (according to medical records) were excluded.

All patients received written information about their participation in the Register and the long-term follow-up, and their verbal and written consent was obtained [27].

**Figure 1:**
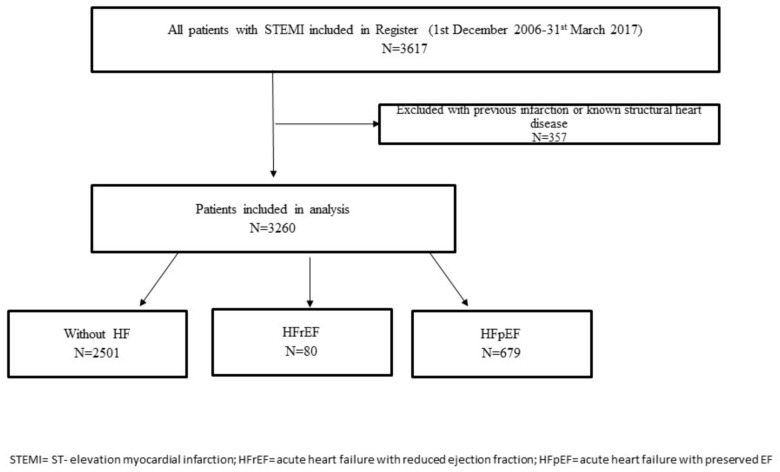
The flowchart of the patient selection.

The flowchart of patient selection is presented in Figure 1.

Coronary angiography, primary PCI, and stenting (or percutaneous optimal balloon angioplasty without stent implantation) of the infarct-related artery (IRA) were performed using the standard technique. The femoral approach was the vascular access in patients hospitalized between 2005 and 2012, while in patients hospitalized between 2013 and 2017, the radial approach was the preferred vascular access. Loading doses of aspirin (300 mg) were administered in all patients before pPCI. Loading doses of clopidogrel (600 mg) or ticagrelor (180 mg) were administered to all patients before pPCI, depending on the current guideline recommendations at the time of the index procedure. Selected patients were also given the glycoprotein (GP) IIb/IIIa receptor inhibitor during the procedure. After pPCI, patients were treated according to the current guidelines.

Demographic, baseline clinical, laboratory, angiographic, and procedural data were collected and analyzed. Baseline kidney function (blood for creatinine analysis was drawn at hospital admission, before iodine contrast administration) was assessed using the Modification of Diet in Renal Disease equation; the value of estimated glomerular filtration rate (eGFR) < 60 mL/min/m^2^ was considered as chronic kidney disease (CKD). Atrial fibrillation (AF) was considered as new-onset if patients had no medical history of previous AF.

An echocardiographic examination was performed in all patients before hospital discharge. The left ventricular EF was assessed according to the biplane method. The value of EF ≥ 50% was considered as preserved EF. Diagnosis of HF (Killip class > 1) during index hospitalization was made in patients with typical symptoms and signs of HF (clinical examination and corroboration by objective evidence of pulmonary and systemic congestion), after excluding any other diseases that can cause the above-described symptoms and signs. Patients were divided into three groups: those without HF, those with HF and reduced EF < 50% (HFrEF), and those with HF and preserved EF ≥ 50% (HFpEF).

Patients were followed up for eight years after the index event. Follow-up data were obtained through telephone interviews and at outpatient visits. We analyzed all-cause mortality.

### 2.2. Ethics

This study was conducted in accordance with the principles of the Helsinki Declaration. The introduction of the Registry was approved by the Ethics Committee of the University of Belgrade Faculty of Medicine (approval number 470/II-4, 21 February 2008).

### 2.3. Statistical Analysis

Categorical variables were expressed as frequency and percentage, and continuous variables were expressed as the median (med), with 25th and 75th quartiles (IQR). The distribution of the numeric data was assessed using the Kolmogorov–Smirnov test. Differences among groups were analyzed using the Man–Whitney U test for continuous variables (data with non-normal distribution) and independent samples *T*-test (for data with normal distribution). The Pearson χ^2^ test was used for analyzing differences between categorical variables. The Kaplan–Meier method was used for constructing the probability curves for eight-year mortality. The difference in mortality rate among patients with HFpEF, with HFrEF, and without HF was tested with the log-rank test. The Cox proportional hazard model (backward method, with *p* < 0.10 for entrance into the model) was used to define univariable and multivariable predictors for the occurrence of eight-year mortality. Two-tailed *p*-values of less than <0.05 were considered statistically significant and two-tailed *p*-values of less than 0.01 as highly statistically significant. For statistical analysis, we used the SPSS statistical software, version 19 (SPSS Inc., Chicago, IL, USA).

## 3. Results

Of the 3260 patients analyzed, 915 (28.1%) were women. The median age of all analyzed patients was 60 (52, 69) years. In-hospital HF was registered in 759 (23.2%) patients. Among the patients with in-hospital HF, 80 (10.5%) patients had HFpEF.

Compared to patients without HF, patients with HFpEF were older. They were more likely to have diabetes mellitus (DM), lower systolic blood pressure at admission, higher heart rate at admission, complete atrioventricular (AV) block, new-onset atrial fibrillation, and post-procedural TIMI flow <3 through the infarct-related artery, but they were less likely to have hyperlipidemia and to be smokers. In-hospital mortality was similar in these two groups of patients. When we compared patients with HFpEF and those with HFrEF, we found that patients with HFpEF were less likely to have multivessel coronary artery disease (on initial angiogram), post-procedural flow TIMI < 3 through the IRA, and baseline chronic kidney disease. Also, patients with HFpEF had a shorter hospital stay and lower in-hospital mortality when compared with patients with HFrEF. Except for a higher percentage of patients with HFrEF who were discharged with diuretic therapy and amiodarone, there was no difference in other therapy at hospital discharge among all the three analyzed groups.

Baseline characteristics, laboratory, angiographic, and procedural characteristics, in-hospital mortality, and therapy at discharge, in patients without HF, patients with in-hospital HFpEF, and patients with in-hospital HFrEF, are presented in Table 1.

At eight-year follow-up, all-cause mortality was registered in a total of 267 (8.2%) patients. Causes of mortality were predominantly cardiovascular in all of the analyzed groups. Non-cardiovascular causes of death (such as cancer, ileus, pneumonia, and dementia) were registered in a total of 28 patients (10.8% of all deaths). After one-year follow-up, significantly higher mortality was recorded in patients with HFpEF compared to those without HF, while over the entire follow-up period, mortality in patients with HFpEF was significantly lower than in those with HFrEF.

Mortality during follow-up is presented in Table 2.

**Figure 2:**
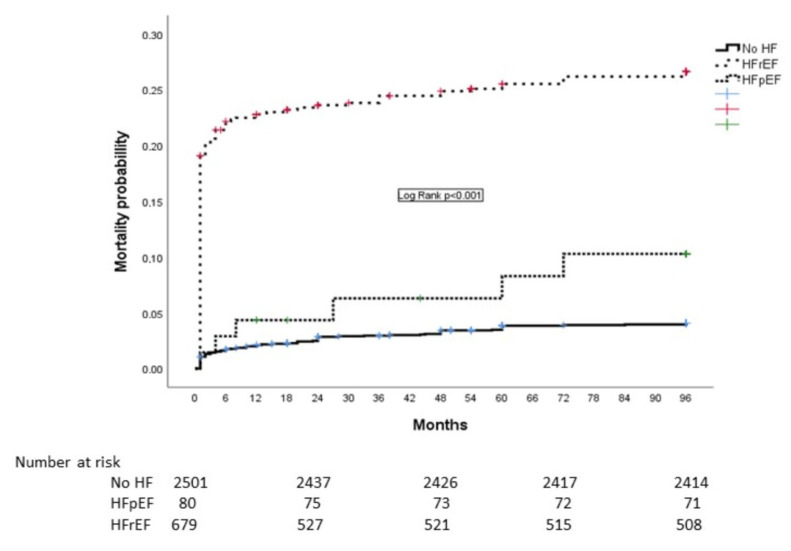
Kaplan Meier curves showing 8-years mortality in patients without AHF, AHFpEF and AHFrEF.

Kaplan–Meier curves showing the probability of mortality during eight-year follow-up are presented in Figure 2.

After adjustment for confounders, both HFpEF and HFrEF were independent predictors of mortality in eight-year follow-up. The negative prognostic impact of HFpEF was less pronounced than the negative prognostic impact of HFrEF. Predictors for the occurrence of eight-year mortality are presented in Table 3.

## 4. Discussion

The results of this study show that the occurrence of heart failure during index hospitalization was recorded in approximately 23% of the analyzed STEMI patients. The predominant cause of in-hospital HF was reduced left ventricular systolic function (i.e., reduced ejection fraction), while 10.5% of patients with in-hospital HF had preserved ejection fraction.

There was no significant difference in short-term mortality (up to one year) between patients with HFpEF and those without HF. However, after one year, patients with HFpEF had significantly higher mortality compared to those without HF, and this difference persisted throughout the follow-up period (eight years).

Additionally, during the entire follow-up period, mortality among patients with HFpEF was significantly lower than in those with HFrEF. HFpEF was an independent predictor of eight-year mortality. The negative prognostic impact of HFpEF on eight-year mortality was weaker than the impact observed in patients with HFrEF.

### 4.1. Patient Baseline Characteristics and the Incidence of HF

The incidence of HFpEF and HFrEF in our patients is generally in keeping with previous findings [4,5,9,18,27]. In some earlier reports, the incidence of HF was higher or lower than in our study [9,11]. The differences in these findings can be explained by the varying populations of patients analyzed. In studies that included both STEMI and non-ST-elevation myocardial infarction (NSTEMI) patients, the incidence of HF was generally lower [5,9]. In studies where myocardial revascularization was not performed in all patients, the incidence of HF was higher compared to our findings [5,11]. Thus, in an analysis of data from the ACTION Registry-GWTG, it was found that as many as 22% of STEMI patients with HF during index hospitalization had preserved left ventricular EF. However, in that study, only about 80% of STEMI patients were treated with primary PCI [5].

The baseline characteristics of our patients with HFpEF are comparable with those found in the literature [9,12,15,18,27]. Compared to patients without HF, it is commonly observed that patients with HFpEF are older and have poorer post-procedural TIMI flow through the infarct-related artery [9,18]. Also, when compared to patients with HFrEF, it is often found that patients with HFpEF had better coronary angiograms and a higher rate of post-procedural TIMI grade 3 flow. The age and risk factors of coronary artery disease did not differ between these two groups in this study, and this is also in keeping with some previous reports [9].

### 4.2. Prognosis in STEMI Patients with HFpEF

Numerous studies have proven that heart failure developing during index hospitalization is a well-known major negative prognostic determinant in STEMI patients [4,12,15], even in patients with preserved EF (which is usually considered as a marker of lower risk). A study by Kim et al. found that pre-discharge development of HF (at admission or during hospitalization) was associated with poorer clinical outcomes during the median follow-up of 60 months compared to patients without HF and those with HF developing after hospital discharge [4].

A study by De Luca et al. found that one-year mortality was eight times higher in STEMI patients who experienced HF during index hospitalization, as compared to STEMI patients without HF [12]. Studies analyzing STEMI patients with HFpEF have shown that short-term mortality in patients with HFpEF complicating STEMI is lower and that the negative prognostic impact of HFpEF is weaker compared to that of HFrEF, which aligns with our findings [5,9,18,27].

In a study by Antonelli et al., the highest in-hospital mortality was observed in patients with systolic HF (EF < 50%), while patients with HF and preserved EF (>50%) had lower in-hospital mortality compared to those with systolic dysfunction, but significantly higher mortality than patients without HF. Moreover, the negative, independent prognostic impact of HF with preserved EF on in-hospital mortality was lower than that of systolic HF, which is consistent with our results [9].

Similar findings were reported in a study by Xu et al., which confirmed that patients with AMI and in-hospital HFpEF had significantly higher in-hospital mortality and more complications compared to patients without HF [18].

Data from the CRUSADE study showed that NSTEMI patients with HFpEF have higher early mortality compared to patients without HF. In this study, 12% of patients had HFpEF, which is slightly higher than in our cohort. However, preserved EF was defined as EF ≥ 40%, meaning that, according to current guidelines, patients with moderately reduced EF (40–49%) were also included in the preserved EF group [27]. Additionally, patients with HF and preserved systolic function (EF > 40%) had approximately two-fold lower in-hospital mortality compared to those with HF and reduced systolic function (EF < 40%) [27].

Unlike the previously cited studies, in our cohort, there was no difference in short-term mortality between the HFpEF group and the group without HF, but a significant difference was observed during long-term follow-up, beginning with one year after the index event.

### 4.3. Mechanisms of HF Development in STEMI Patients

Although coronary artery disease is considered one of the most important causes of HFrEF, recent data show that a large percentage of patients with AMI develop HFpEF [28]. HF complicating AMI is a result of complex and unbalanced structural, hemodynamic, neurohumoral interactions, and inflammation [9,10,17,18,22,29,30]. Ischemia and myocardial necrosis cause both systolic and diastolic dysfunction because ventricular diastole is an active process that consumes oxygen [9]. The percentage of patients with diastolic dysfunction following STEMI is around 20–30% [22]. Also, in the contemporary pPCI era, smaller infarct (necrotic) size usually does not reduce systolic function but may lead to diastolic dysfunction [28]. Inflammation is a very important mechanism that leads to cardiac dysfunction and adverse remodeling, and it is also a significant prognostic marker in STEMI patients. In all STEMI patients, there is the initial inflammatory phase (caused by myocardial cell necrosis) followed by an anti-inflammatory phase that helps myocardial scar formation. Dysregulated inflammation with delayed anti-inflammatory phase is associated with extensive cardiac myocyte death and impaired systolic and diastolic function of the remaining myocytes [29,30]. Also, ischemia-reperfusion injury after opening of an infarct related artery is partially driven by inflammation through multiple interacting pathways. Even without extensive necrosis, ischemia-reperfusion injury with inflammatory response, stunned and hibernating myocardium also has relaxation/diastolic dysfunction, which is usually transitory and causes (transitory) in-hospital HF in patients with preserved EF [3,9,18]. According to some authors, STEMI patients with preserved EF are usually less likely to receive drugs that positively affect their prognosis and possible further myocardial remodeling after STEMI (e.g., beta blockers and/or ACE inhibitors, etc.) [28]. This finding may explain the poorer long-term prognosis of STEMI patients with HFpEF compared to STEMI patients without HF. In this study, there was no significant difference in the prescription of the said therapy at discharge among the analyzed patients. Therefore, the poorer survival of our STEMI patients with HFpEF cannot simply be attributed to the insufficient implementation of guideline-directed therapy.

### 4.4. Clinical Implications

Despite having a similar in-hospital outcome as patients without HF, STEMI patients with in-hospital HFpEF are at higher risk of mortality during long-term follow-up, as compared to patients without in-hospital HF. Bearing this in mind, STEMI patients with HFpEF should be considered to have an increased risk of mortality during long-term follow-up. Therefore, they require a more individualized approach, including closer follow-up, even beyond the first year following the index event and tailored treatment approach. There should be a strong emphasis on strict control of risk factors for coronary artery disease and the development of HFpEF in general (primarily hypertension and diabetes mellitus), as well as on the prevention and management of obesity [24]. In addition to lowering cholesterol level, statins exert anti-inflammatory effects of cells involved in atherosclerosis and high-dose statin should be prescribed in all STEMI patients, especially in those with HF [29]. Also, introducing sodium glucose transporter inhibitors-2 (SGLT-2 inhibitors) in patients with in-hospital HFpEF should be considered regardless of the presence of DM [31].

### 4.5. Study Limitations

This study should be viewed in the context of its limitations. The study is unicentric and observational, but it is controlled, prospective, and has included consecutive patients, limiting possible selection bias. Patients with cardiogenic shock at admission were excluded from our Register. We did not use other measures for determining systolic function, such as myocardial deformation imaging. However, numerous clinical trials have used EF to stratify patients, demonstrating its benefits in determining the outcome [Ng, Yilmaz]. Data on echocardiographic parameters used for assessing diastolic function (other than E/A ratio and left atrial diameter) were not available in all patients in our Register. There were no data on follow-up echocardiographic examinations to show whether there had been a certain degree of recovery or deterioration in the myocardial contractility. Natriuretic peptides were not determined in all patients in our Register. New-generation oral antidiabetic drugs, such as sodium glucose transporter-2 (SGLT-2) inhibitors, were unavailable to our patients at the time of patient inclusion, which may have influenced long-term prognosis in those with HFpEF. This study was not designed to evaluate whether changing pharmacological treatment during follow-up would impact the long-term outcome in the analyzed patients.

## 5. Conclusions

Around a quarter of the patients with STEMI developed in-hospital HF, predominantly caused by reduced left ventricular systolic function. However, 10% of STEMI patients with in-hospital HF had preserved EF. STEMI patients with HFpEF had similar in-hospital mortality and significantly higher long-term mortality, as compared to STEMI patients without HF. Short- and long-term mortality was significantly lower in patients with HFpEF compared to patients with HFrEF. Development of in-hospital HFpEF in STEMI patients was an independent predictor for long-term mortality. However, the negative prognostic impact of HFpEF was weaker when compared to the impact of in-hospital HFrEF.

## Figures and Tables

**Table 1:** Baseline clinical, laboratory, angiographic, procedural characteristics, therapy at discharge, and intrahospital mortality of the study patients.

Characteristics	Without HF N = 2501	HFpEF N = 80	*p*-Value *	HFrEF N = 679	*p*-Value **
Age, years med (IQR)	58 (45, 75)	65 (45, 85)	<0.001	64 (46, 82)	0.828
Female, n (%)	667 (26.6)	22 (27.5)	0.453	226 (33.2)	0.064
BMI, med (IQR)	26.3 (22.1, 30.5)	25.5 (20.1, 31.5)	0.967	26.8 (21.8, 31.2)	0.897
Previous angina, n (%)	174 (7)	8 (10)	0.173	57 (8.5)	0.436
Previous stroke, n (%)	79 (3.2)	4 (5)	0.254	45 (6.6)	0.234
Diabetes, n (%)	427 (17.1)	19 (23.7)	0.038	203 (29.8)	0.535
Hypertension, n (%)	1652 (66.1)	51 (63.8)	0.730	488 (71.8)	0.335
HLP, n (%)	1555 (62.2)	32 (40)	0.003	380 (55.9)	0.064
Smoking, n (%)	1432 (57.3)	30 (37.5)	0.012	279 (41)	0.792
Family history, n (%)	874 (34.5)	24 (30)	0.951	182 (26.8)	0.238
Pain duration, hours, med (IQR)	2.5 (2, 3.5)	2.5 (1.5, 3.5)	0.678	3 (1.5, 4.5)	0.444
Systolic BP at admission, med (IQR)	140 (110, 150)	120 (110, 150)	0.022	130 (110, 155)	0.975
Heart rate at admission med (IQR)	76 (70, 85)	100 (80, 116)	<0.001	90 (70, 98)	0.063
New-onset atrial fibrillation, n (%)	104 (4.2)	8 (9.7)	0.021	114 (16.7)	0.121
Complete AV block, n (%)	85 (3.4)	7 (8.7)	0.004	54 (7.9)	0.808
KIllip class II n (%)	N/A	78 (97.5)	N/A	509 (74.9)	<0.001
Killip class III (%)	N/A	2 (2.5)	N/A	111 (16.3)	<0.001
KIllip class IV (%) ***	N/A	0	N/A	59 (8.6)	<0.001
Multivessel disease, n (%)	1323 (52.3)	46 (56.9)	0.480	471 (69.3)	0.031
LM stenosis, n (%)	134 (5.4)	5 (6.2)	0.553	57 (8.4)	0.502
LAD as culprit vessel, n (%)	819 (32.7)	30 (37.5)	0.234	466 (68.7)	<0.001
RCA as culprit vessel, n (%)	1230 (49.1)	32 (40)	0.123	128 (18.9)	<0.001
Cx as culprit vessel, n (%)	318 (12.7)	9 (11.2)	0.545	58 (8.6)	<0.001
Stent implanted, n (%)	2384 (95.3)	71 (88.9)	0.013	603 (88.9)	0.989
Postprocedural flow TIMI <3, n (%)	55 (2.2)	5 (6.2)	0.008	86 (12.7)	<0.001
Acute stent thrombosis, n (%)	26 (1.1)	1 (1.2)	0.336	11 (1.6)	0.883
Peak CK MB U/L med (IQR)	1673 (1430, 5435)	2819 (1645, 4578)	0.096	2883 (1435, 6789)	0.009
Peak Troponin I ng/mL, med (IQR)	29 (14.3, 105)	40 (30, 130,1)	0.456	52.8 (34.6, 159)	0.051
WBC at admission × 10^9^, med (IQR)	11.1 (6.8–15.2)	12.1 (7.7–16.4)	0.081	12.3 (7–16.2)	0.549
Hemoglobin at admission g/L, med (IQR)	143 (123, 163)	142 (126, 156)	0.061	139 (117, 152)	0.678
Baseline CKD, n (%)	1711 (68.4)	52 (65.2)	0.108	517 (76.1)	0.074
EF, med (IQR)	50 (40, 61)	55 (50, 60)	0.070	40 (30, 45)	<0.001
LVEDD, med (IQR)	5.5 (5.1, 5.8)	5.5 (5, 5.7)	0.130	5.7 (5.3, 6.7)	0.018
E/A ratio	0.85 ± 0.39	0.79 ± 0.28	0.153	0.96 ± 0.65	0.014
Therapy at discharge ****					
Beta blockers, n (%)	2222 (88.9)	65 (81.2)	0.181	481 (70.8)	0.178
ACE inhibitors, n (%)	2056 (82.2)	62 (77.5)	0.204	462 (68)	0.417
Statin, n (%)	2206 (88.2)	66 (82.5)	0.559	481 (70.9)	0.447
Diuretic, n (%)	259 (10.3)	4 (5)	<0.001	236 (34.8)	<0.001
Calcium antagonist, n (%)	88 (3.5)	4 (5)	0.404	15 (2.2)	0.184
Amiodarone, n (%)	64 (2.6)	1 (1.2)	0.074	25 (3.7)	0.065
Length of hospital stay, days, med (IQR)	7 (5, 9)	7 (6, 9)	0.830	9 (7, 13)	<0.001
In-hospital mortality, n (%)	12 (0.5)	1 (1.2)	0.282	119 (17.5)	<0.001

N/A = not applicable. * *p*-value for patients without HF and patients with HFpEF; ** *p*-value for patients with HFpEF and patients HFrEF; *** during hospitalization, patients with cardiogenic shock are excluded from this Register; **** all patients were on Aspirin and P2Y12-receptor blocker at discharge. HF = heart failure; HFpEF = heart failure with preserved ejection fraction; HFrEF = heart failure with reduced ejection fraction; med = median; IQR = interquartile range; BMI = body mass index; AV = atrioventricular; HLP = hyperlipidemia; BP = arterial blood pressure; EF = left ventricular ejection fraction; LM = left main artery; LAD = left anterior descending coronary artery; Cx = circumflex coronary artery; RCA = right coronary artery; LVEDD = left ventricular end diastolic diameter; WBC = white blood cell count at admission; CKD = chronic kidney disease; CK MB = creatine kinase MB isoform; E/A = transmitral inflow ratio.

**Table 2:** Mortality during follow-up in patients without HF, patients with HFpEF, and patients with HFrEF.

	Without HF N = 2501	HFpEF N = 80	*p*-Value *	HFrEF N = 679	*p*-Value **
1 month mortality	19 (0.7)	1 (1.1)	0.282	124 (18.2)	<0.001
1-year mortality	53 (2.1)	4 (5)	0.016	150 (22.1)	<0.001
8-year mortality	87 (3.5)	9 (11.2)	<0.001	171 (25.1)	<0.001

* *p*-value for patients without HF and patients with HFpEF; ** *p*-value for patients with HFpEF and patients with HFrEF; HFpEF = heart failure with preserved EF; HFrEF = heart failure with reduced EF.

**Table 3:** Independent predictors for 8-year mortality (Cox regression model) in all analyzed patients.

	Univariable Analysis		Multivariable Analysis	
	HR (95%CI)	*p*-Value	HR (95%CI)	*p*-Value
Age, years	1.07 (1.06–1.08)	<0.001	1.05 (1.04–1.06)	<0.001
In-hospital HF	8.03 (5.78–11.95)	<0.001	4.78 (3.21–7.12)	<0.001
HFrEF	7.85 (6.05–10.18)	<0.001	4.89 (3.19–6.42)	<0.001
HFpEF	2.43 (1.06–5.57)	0.013	1.85 (1.26–4.25)	0.012
Post-procedural flow TIMI < 3	6.47 (4.79–8.75)	<0.001	2.84 (2.06–3.92)	<0.001
New-onset AF	4.39 (3.28–5.87)	<0.001	1.47 (1.07–2.01)	0.017
Multivessel disease	2.58 (2.02–3.95)	<0.001	1.39 (1.07–1.80)	0.014
Anaemia at admission	2.17 (1.95–3.74)	<0.001		
Diabetes	2.11 (1.62–2.73)	<0.001		
Baseline CKD	1.46 (1.11–1.67)	0.051		
LAD culprit vessel	1.46 (1.04–1.68)	0.050		
Male gender	1.42 (1.08–1.57)	0.053		

AF = atrial fibrillation; HF = heart failure; HFrEF = heart failure with reduced ejection fraction; HFpEF = heart failure with preserved ejection fraction; CKD = chronic kidney disease; LAD = left anterior descendent coronary artery.

## Data Availability

The data presented in this study are available on request from the corresponding author due to ethical reasons.
